# RNA metabolic regulation plays diverse roles in nutrient-dependent seedling growth in Arabidopsis

**DOI:** 10.5511/plantbiotechnology.25.0902a

**Published:** 2026-03-25

**Authors:** Kodai Ishibashi, Misato Ohtani

**Affiliations:** 1 Department of Integrated Biosciences, Graduate School of Frontier Science, The University of Tokyo, Tsukuba, Ibaraki 305-8572, Japan

**Keywords:** nutrients, pre-mRNA splicing, RNA degradation, seedling growth, sucrose

## Abstract

Plants continuously respond to changes in nutrient availability by adjusting their growth and development. However, the role of RNA metabolism in this process is unclear. We performed seedling growth assays using *Arabidopsis thaliana* mutants defective in RNA metabolism. When grown on full-strength Murashige and Skoog (MS) medium containing 1% sucrose, all mutants had shorter primary roots than the wild type, suggesting the importance of RNA metabolic regulation for seedling growth under nutrient-rich conditions. Primary root growth exhibited distinct responses to the presence of sucrose in the following mutants: *ccr4a ccr4b*, with defects in the deadenylases CARBON CATABOLITE REPRESSOR4a (CCR4a) and CCR4b, which function in poly(A) tail degradation; *mtr4-2*, with a mutation in the exosome co-factor mRNA TRANSPORT4 (MTR4), which is required for 3′–5′ RNA degradation; and *rid1-1*, with a defect in the RNA helicase ROOT INITIATION DEFECTIVE1 (RID1), which functions in pre-mRNA splicing. Whereas the *ccr4a ccr4b* seedlings did not exhibit sucrose-dependent changes in root growth, the *mtr4-2* and *rid1-1* seedlings exhibited more pronounced growth inhibition in response to a lack of sucrose and reduced MS salt and vitamin concentrations, respectively, compared to the wild type. When grown on 0.1×MS medium without sucrose, *upf3-1* seedlings, which lack functional UP-FRAMESHIFT3 (UPF3), a component of nonsense-mediated mRNA decay (NMD), were larger than the wild type, suggesting the importance of NMD in regulating seedling growth under nutrient-limited conditions. Therefore, different RNA metabolic pathways play distinct roles in the nutrient-dependent regulation of plant growth, adjusting plant fitness to different environments.

Eukaryotic mRNAs are the targets of multiple types of metabolic regulation, including 5′ capping, pre-mRNA splicing, poly(A) tailing, changes in their subcellular localization, and degradation. RNA quality control mechanisms, such as nonsense-mediated mRNA decay (NMD), remove mRNA molecules with premature termination codons (Ohtani and Wachter 2019). Molecular and genetic analyses of Arabidopsis (*Arabidopsis thaliana*) mutants have revealed that the metabolic regulation of RNA is important for abiotic and biotic stress responses in plants (Matsui et al. 2019) and for plant responses to nutrient availability. For example, several microRNAs (miRNAs) function in the uptake and translocation of nutrients by regulating the expression of nutrient transporter genes and/or transcription factor genes that regulate plant architecture and symbiosis (Chien et al. 2017; Sexauer et al. 2023).

The Arabidopsis *upf1-1* mutant, which is defective in UP-FRAMESHIFT1 (UPF1, also known as LOW-LEVEL BETA-AMYLASE1 [LBA1]), the core factor of the NMD pathway, exhibits sugar-dependent growth abnormalities, such as hypersensitivity to glucose and resistance to mannose, during germination and seedling growth (Yoine et al. 2006). Additionally, a double mutant of *CARBON CATABOLITE REPRESSOR4A* (*CCR4A*) and *CCR4B* is more tolerant than the wild type to growth on medium containing high sucrose levels (Suzuki et al. 2015). Moreover, Nishida and co-authors documented the dynamics of alternative mRNA splicing as a function of nutrient status based on transcriptome deep sequencing (RNA-seq) of transcripts from the roots of Arabidopsis seedlings grown on medium lacking 12 nutrients (Nishida et al. 2017). The alternative splicing of *MYB59* transcripts under potassium-limited conditions is thought to regulate the expression of the potassium transporter gene *NRT1/PTR FAMILY7.3* (*NPF7.3*) in shoots, thus affecting potassium levels in this tissue (Enomoto et al. 2023). These findings strongly suggest that RNA metabolic regulation is fundamental to plant nutrient responses. However, to date, no studies have compared the responses of multiple RNA metabolism–related mutants to changes in nutrient status under the same growth conditions.

To bridge this gap, we investigated the growth of wild-type Arabidopsis and RNA metabolism-related mutant seedlings under various nutrient conditions. Based on the literature, we selected the RNA degradation-related mutants *ccr4a ccr4b*, which is defective in poly(A) degradation (Suzuki et al. 2015), and *mtr4-2*, which lacks mRNA TRANSPORTER4 (MTR4), an RNA exosome component involved in 3′–5′ RNA degradation (Lange et al. 2011, 2014); the NMD-deficient mutant *upf3-1* (Hori and Watanabe 2005); the pre-mRNA defective mutants *shoot redifferentiation defective2-1* (*srd2-1*) (Ohtani and Sugiyama 2005) and *root initiation defective1-1* (*rid1-1*) (Ohtani et al. 2013); and the *regulator of fatty acid composition3-2* (*rfc3-2*) mutant, which lacks a functional plastid-localized ribosomal S6-like protein (Horiguchi et al. 2003) (Supplementary Table S1).

We sowed seeds from the above mutants, along with seeds from wild-type Arabidopsis accessions Col-0 (for *ccr4a ccr4b*, *mtr4-2*, and *upf3-1*) and Landsberg *erecta* (L*er*, for *rfc3-2*, *rid1-1*, and *srd2-1*), on medium containing Murashige and Skoog salts and vitamins (MS hereafter) (Duchefa Biochem, Haarlem, Netherlands) (Murashige and Skoog 1962) supplemented with sucrose (Suc) (WAKO, Osaka, Japan), adjusted to pH 5.7 with 0.05% (w/v) 2-(*N*-morpholino) ethanesulfonic acid (Nacalai Tesque, Kyoto, Japan), and solidified with 1.5% (w/v) agar (WAKO). We tested four growth conditions: 1) full-strength MS with 1% (w/v) Suc; 2) full-strength MS without Suc; 3) half-strength (0.5×) MS without Suc; and 4) one-tenth-strength (0.1×) MS without Suc. We grew the seedlings under a 16-h light/8-h dark photoperiod at 22°C for 14 days before scanning the seedlings and measuring root length using ImageJ Fiji software [https://imagej.net/software/fiji/downloads (Accessed July 1, 2025)]. We performed all growth tests at least three times, and in each experiment, more than three seedlings per genotype were grown on the same plate as the wild type. Representative replicates are shown in the figures.

The growth of wild-type seedlings was strongest under nutrient-rich conditions, specifically on full-strength MS medium supplemented with 1% (w/v) Suc (MS+1%Suc) (Figures 1, 2, Supplementary Figure S1). In wild-type seedlings, the absence of Suc (MS+0%Suc and 0.5MS+0%Suc) resulted in a 36% and 18% reduction in average primary root length, respectively, compared to nutrient-rich conditions (Figure 2A). The most pronounced growth inhibition of wild-type seedlings occurred when the concentration of MS salts and vitamins was reduced to one-tenth and no Suc was added (0.1MS+0%Suc), resulting in markedly smaller roots and shoots, with roots that were 92% shorter than those of plants grown on MS+1%Suc (Figures 1, 2, Supplementary Figure S1). A lack of Suc appeared to have a greater effect on seedling growth in L*er* (an average of 46% shorter roots) than in Col-0 (26% shorter roots) (Figures 1, 2, Supplementary Figure S1), suggesting that the response to Suc during early development is accession-dependent. All tested mutants had shorter primary roots when grown on MS+1%Suc than the respective wild-type plants (Figure 2A, Supplementary Figure S1). Thus, RNA metabolism is important for early seedling development under nutrient-rich conditions.

**Figure 1:**
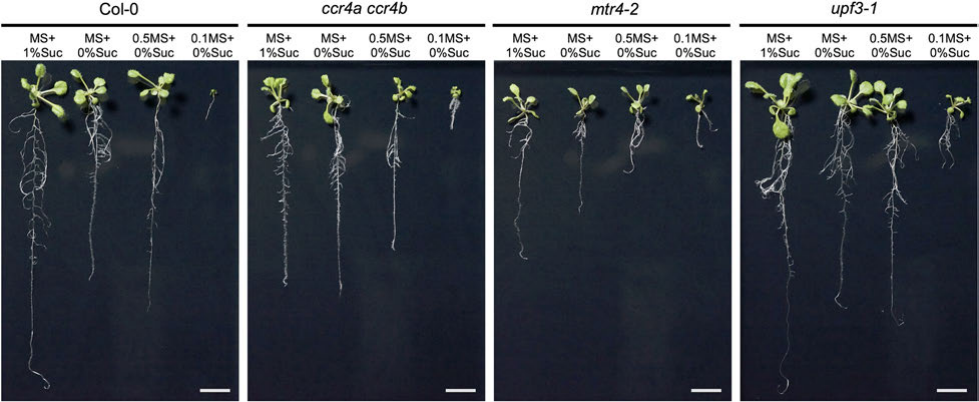
Figure 1. Representative photographs of 14-day-old seedlings of the wild type (Col-0) and RNA metabolism-related mutants grown under different nutrient conditions. MS+1%Suc, full-strength MS medium with 1% (w/v) Suc; MS+0%Suc, full-strength MS medium without Suc; 0.5MS+0%Suc, half-strength (0.5×) MS medium without Suc; 0.1MS+0%Suc, one tenth-strength (0.1×) MS medium without Suc. The plants were grown on plates placed vertically, and typical individuals from each line were transferred to the new plate and photographed. Scale bars, 1 cm.

**Figure 2:**
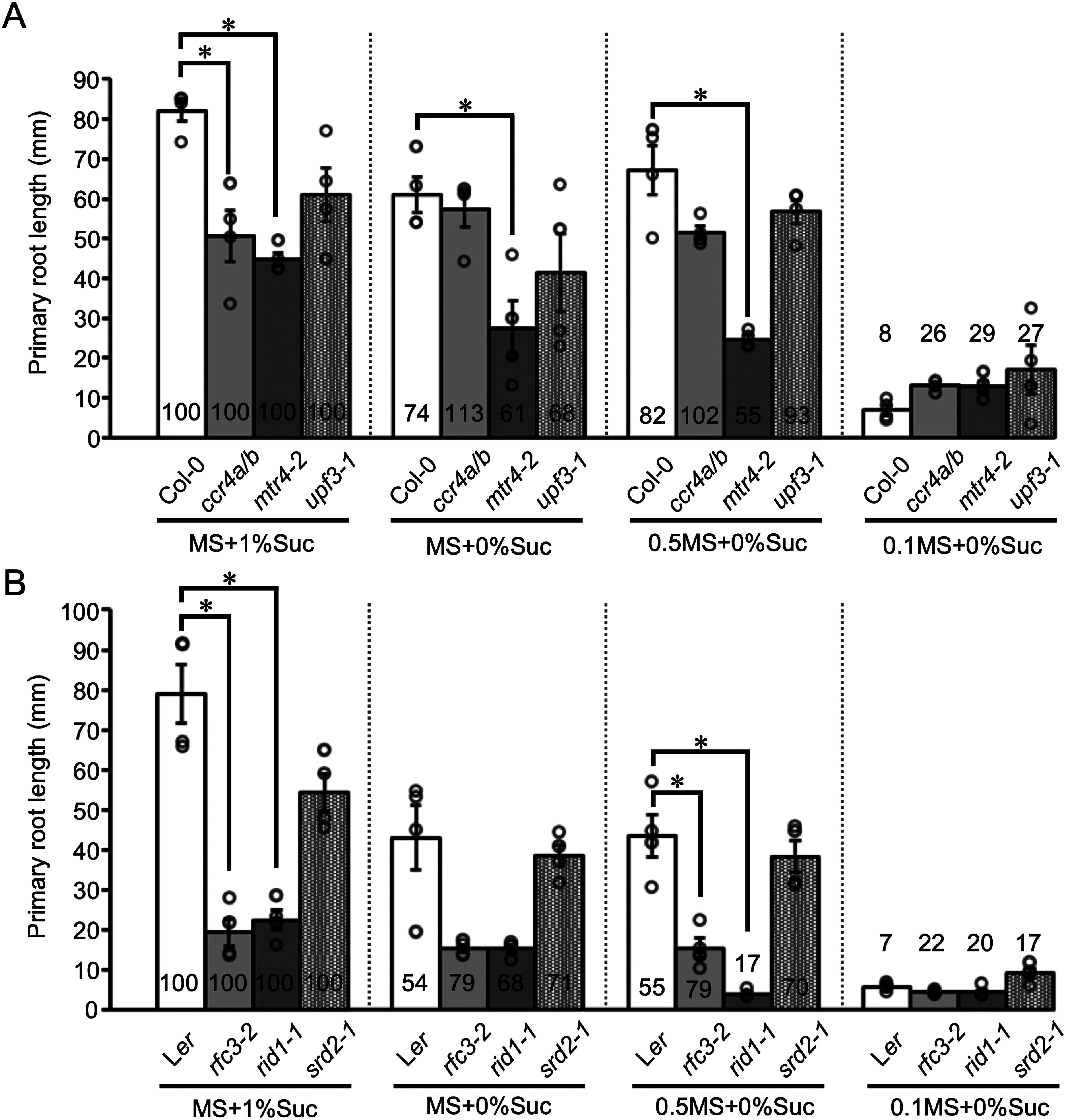
Figure 2. Primary root lengths of 14-day-old seedlings of the wild type and RNA metabolism-related mutants grown under different nutrient conditions. (A) Primary root length for seedlings of Col-0 and RNA metabolism-related mutants in the Col-0 background. (B) Primary root length for seedlings of L*er* and RNA metabolism-related mutants in the L*er* background. MS+1%Suc, full-strength MS medium with 1% (w/v) Suc; MS+0%Suc, full-strength MS medium without Suc; 0.5MS+0%Suc, half-strength (0.5×) MS medium without Suc; 0.1MS+0%Suc, one tenth-strength (0.1×) MS medium without Suc. Values are expressed as means ± standard error (SE; *n*=4). Asterisks indicate significant differences from the wild type (Student’s *t*-tests with Bonferroni correction, *p*<0.05). The values at the base of each bar represent the percentage of primary root length under each nutrient condition normalized to that under MS+1%Suc conditions, which was set to 100.

The growth of all mutant seedlings was influenced by the nutrient status. The roots of 14-day-old *ccr4a ccr4b* seedlings were shorter than wild-type roots when grown on MS+1%Suc, but the roots were the same length in the absence of Suc, reaching a length comparable to that of the wild type on MS+0%Suc (Figures 1, 2A). As CCR4A and CCR4B were previously shown to be involved in Suc metabolism (Suzuki et al. 2015), we suggest that the growth inhibition observed in *ccr4a ccr4b* in the presence of Suc may reflect abnormal RNA turnover of Suc metabolism-related genes. By contrast, the growth of *mtr4-2* seedlings was more strongly inhibited in response to low nutrient levels than that of the wild type. Indeed, the roots of *mtr4-2* seedlings were significantly reduced on 0.5MS+0%Suc (45% shorter on 0.5MS+0%Suc than on MS+1%Suc), whereas the roots of wild-type plants were 18% shorter on 0.5MS+0%Suc compared to MS+1%Suc (Figure 2A, Supplementary Figure S2). As MTR4 functions in ribosomal RNA processing (Lange et al. 2011, 2014), and Suc levels can influence the translation of specific mRNAs (Gamm et al. 2014), the strong sensitivity of *mtr4-2* to the lack of Suc might be related to a reduction in protein translation activity. Among the RNA degradation-related mutants we assessed, *upf3-1* demonstrated a unique phenotype: the roots of *upf3-1* seedlings showed no significant differences from the wild type when grown on MS+1%Suc, MS+0%, or 0.5MS+0%. However, under the poorest nutrient conditions (0.1MS+0%), *upf3-1* seedlings fared better than the wild type, particularly in terms of shoot growth (Figure 2A, Supplementary Figure S3). UPF3 is involved in NMD, a quality-based mRNA degradation system (Ohtani and Wachter 2019). Together, these observations suggest that the NMD pathway is not closely involved in Suc-dependent growth regulation but rather contributes to growth regulation under poor nutrient conditions.

The growth of the *rfc3-2* mutant, particularly its roots, was severely inhibited (Figure 2B, Supplementary Figure S1), which is consistent with previous reports of abnormal root development and growth under high Suc conditions in this mutant (Horiguchi et al. 2003; Nagashima et al. 2020). Under the conditions tested, the roots of *rfc3-2* seedlings were consistently much shorter than those of the wild type (Figure 2B, Supplementary Figure S1), reflecting the finding that RFC3 plays important roles in plastid function in roots (Horiguchi et al. 2003). We observed a similar, severe level of growth inhibition in *rid1-1*, a mutant defective in an RNA helicase involved in pre-mRNA splicing (Ohtani et al. 2013). The shorter root phenotype of *rid1-1* was more pronounced on 0.5MS+0%Suc than on the other media examined (Figure 2B, Supplementary Figure S1). Thus, *rid1-1* appears to be hypersensitive to lower levels of MS salts and vitamins. The roots of *srd2-1* seedlings, which harbor a mutation in the gene encoding another pre-mRNA splicing-related factor, were not hypersensitive to low nutrient status (Figure 2B, Supplementary Figure S1), suggesting that nutrient response phenotypes differ among pre-mRNA splicing mutants, possibly reflecting their respective target transcripts and distinct modes of action in pre-mRNA splicing.

To test whether the absence of Suc altered seedling growth by changing the osmotic pressure of the medium, we grew seedlings on germination medium containing full-strength MS with 0.75% (w/v) mannitol (WAKO), which has the same osmotic pressure as 1% (w/v) Suc (i.e., MS+Man). For Col-0 and L*er*, as well as all mutants in the Col-0 background, there was no significant difference in primary root length between seedlings grown on MS+0%Suc and MS+Man. By contrast, root growth was significantly inhibited in the pre-mRNA splicing–related mutants *rid1-1* and *srd2-1* on MS+Man (Supplementary Figure S4). Perhaps in pre-mRNA splicing mutants, the osmotic pressure imposed by 1% (w/v) Suc is sensed as stress without activating Suc signaling pathways, resulting in severe growth inhibition. Indeed, seedlings harboring a mutation in *SNW/SKI-INTERACTING PROTEIN* (*SKIP*), which encodes a core component of the spliceosome, are highly sensitive to osmotic stress (Feng et al. 2015). Taken together, these findings indicate that different pre-mRNA splicing factors play diverse roles in nutrient responses, as demonstrated by *rid1-1* and *srd2-1*, but may also be involved in regulating the response to stress (e.g., osmotic stress) induced by nutrient conditions.

Finally, we examined how nutrient status affects the expression levels and/or alternative splicing of *CCR4A*, *CCR4B*, *MTR4*, *UPF3*, *RFC3*, *RID1*, and *SRD2* based on RNA-seq data published by Nishida et al. (2017). The relative expression levels were not significantly different for any of these genes under the nutrient conditions examined (Supplementary Figure S5A). We only observed significant changes in alternative splicing in response to nitrogen limitation for *UPF3*, with a 0.72% difference in the relative abundance of a specific alternatively spliced mRNA isoform between control and nitrogen-free conditions (Supplementary Figure S5B). Thus, the expression levels and alternative splicing patterns of the tested genes were stable in response to various nutrient conditions, indicating that the expression levels of these genes are not direct targets of the nutrient response in plants.

In conclusion, we clarified the differential roles of RNA metabolic regulation in nutrient-dependent seedling growth in Arabidopsis. The regulation of RNA degradation is crucial for regulating Suc-dependent growth, and mRNA quality control in particular contributes to growth inhibition under nutrient-poor conditions. In *upf3-1*, the abnormal regulation of Suc-dependent gene expression (Yoine et al. 2006) and the altered response to salt stress (Vexler et al. 2016), pathogen treatment (Jeong et al. 2011), and exogenous auxin treatment (Chiam et al. 2019) were reported. Such abnormalities in *upf3-1* are partly attributed to the accumulation of abnormal splicing variants of key regulatory gene mRNAs (Chiam et al. 2019; Jeong et al. 2011). Thus, the growth acceleration under nutrient-poor conditions in *upf3-1* would be also caused by the abnormal regulation of mRNAs for growth regulator genes. Nutrient-dependent growth differed among pre-mRNA splicing mutants, likely reflecting how different target transcripts are subject to alternative splicing. However, the regulation of pre-mRNA splicing is critical for regulating plant growth in response to the osmotic stress imposed by nutrient conditions. Since the regulation of RNA metabolism affects responses to various stresses and photoperiods (Matsui et al. 2019) in addition to nutrient status, it may serve as a molecular hub that integrates external information and improves overall plant fitness as a function of the environment. In addition, the results of the present study provide fundamental insights into how growth medium conditions during the aseptic culture of plants—a critical method in plant biotechnology—may differentially modulate RNA metabolism and thus influence plant growth and development. Our results may lead to the development of better plant culture media.

## Abbreviations

CCR4: CARBON CATABOLITE REPRESSOR4
Man: mannitol
MS: Murashige and Skoog
MTR4: mRNA TRANSPORTER4
RFC3: REGULATOR OF FATTY ACID COMPOSITION3
RID1: ROOT INITIATION DEFECTIVE1
SRD2: SHOOT REDIFFERENTIATION DEFECTIVE2
Suc: sucrose
UPF3: UP-FRAMESHIFT 3

## Accession numbers

Sequence data from this article can be found in the GenBank/EMBL libraries under the following accession numbers: *CCR4A* (At3g58560), *CCR4B* (At3g58580), *MTR4* (At1g59760), *RFC3* (At3g17170), *RID1* (At1g26370), *SRD2* (At1g28560), *UPF3* (At1g33980).

## Description of supplementary files

Supplementary Table S1. The mutant lines used for this study.

Supplementary Figure S1. Representative photographs of 14-day-old seedlings of the wild type (L*er*) and RNA metabolism-related mutants grown under different nutrient conditions.

Supplementary Figure S2. Primary root lengths of 14-day-old seedlings of the wild type (Col-0) and *mtr4-1* grown under different nutrient conditions.

Supplementary Figure S3. Representative photographs of 14-day-old seedlings of the wild type (Col-0) and the *upf3-1* mutant grown on medium containing 0.1×MS without sucrose.

Supplementary Figure S4. Primary root length of 14-day-old seedlings of the wild type (L*er*) and the *rid1-1* and *srd2-1* mutants grown under different nutrient conditions.

Supplementary Figure S5. Changes in expression levels and alternative splicing of *CCR4A*, *CCR4B*, *MTR4*, *UPF3*, *RFC3*, *RID1*, and *SRD2* in response to the depletion of different nutrients.
