# Supplementary material for: RNA metabolic regulation plays diverse roles in nutrient-dependent seedling growth in Arabidopsis
Source: Plant Biotechnol (Tokyo). 2026 Mar 25;43(1):133–7. doi: 10.5511/plantbiotechnology.25.0902a (PMC13170810; doi:10.5511/plantbiotechnology.25.0902a)
Supplement: Supplementary Data [file plantbiotechnology-43-1-25.0902a-s001.pdf]

Supplementary Table S1. The mutant lines used for this study.

Name of mutant	Type of mutation	Refercence
ccr4a ccr4b	***ccr4a*** **; T-DNA insertion,** ***ccr4b*** **; RNAi**	**Suzuki et al. 2015**
mtr4-2	**T-DNA insertion**	**Lange et al. 2011**
upf3-1	**T-DNA insertion**	**Hori et al. 2005**
srd2-1	**Point mutation**	**Ohtani and Sugiyama 2005**
rid1-1	**Point mutation**	**Ohtani et al. 2013**
rfc3-2	**Point mutation**	**Horiguchi et al. 2003**

Hori K, Watanabe Y (2005) UPF3 suppresses aberrant spliced mRNA in Arabidopsis. *Plant J* 43: 530-540

Horiguchi G, Kodama H, Iba K (2003) Mutations in a gene for plastid ribosomal protein S6-like protein reveal a novel developmental process required for the correct organization of lateral root meristem in Arabidopsis. *Plant J* 33: 521-529

Lange H, Sement FM, Gagliardi D (2011) MTR4, a putative RNA helicase and exosome co-factor, is required for proper rRNA biogenesis and development in *Arabidopsis thaliana*. *Plant J* 68: 51-63

Ohtani M, Sugiyama M (2005) Involvement of SRD2-mediated activation of snRNA transcription in the control of cell proliferation competence in Arabidopsis. *Plant J* 43: 479-490

Ohtani M, Demura T, Sugiyama M (2013) Arabidopsis root initiation defective1, a DEAH-box RNA helicase involved in pre-mRNA splicing, is essential for plant development. *Plant Cell* 25: 2056-2069

Suzuki Y, Arae T, Green PJ, Yamaguchi J, Chiba Y (2015) AtCCR4a and AtCCR4b are Involved in determining the poly(A) length of granule-bound starch synthase 1 transcript and modulating sucrose and starch metabolism in *Arabidopsis thaliana*. *Plant Cell Physiol* 56: 863-874

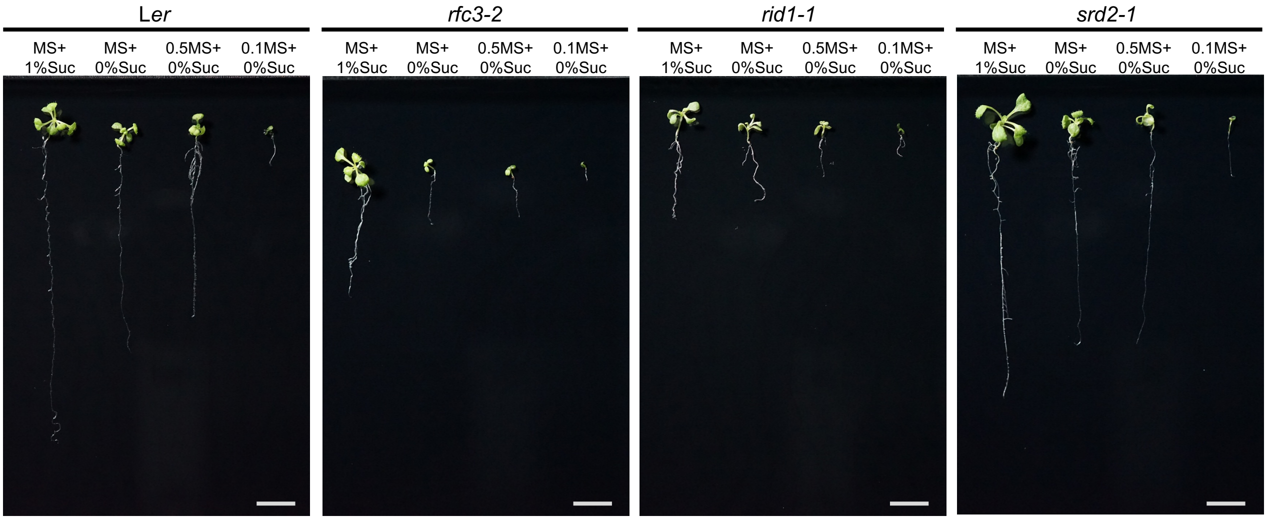

Supplementary Figure S1. Representative photographs of 14-day-old seedlings of the wild type (L*er*) and RNA metabolism–related mutants grown under different nutrient conditions. MS+1%Suc, full-strength MS medium with 1% (w/v) Suc, MS+0%Suc; full-strength MS medium without Suc; 0.5MS+0%Suc, half-strength (0.5×) MS medium without Suc; 0.1MS+0%Suc, one tenth–strength (0.1×) MS medium without Suc. Scale bars, 1 cm.

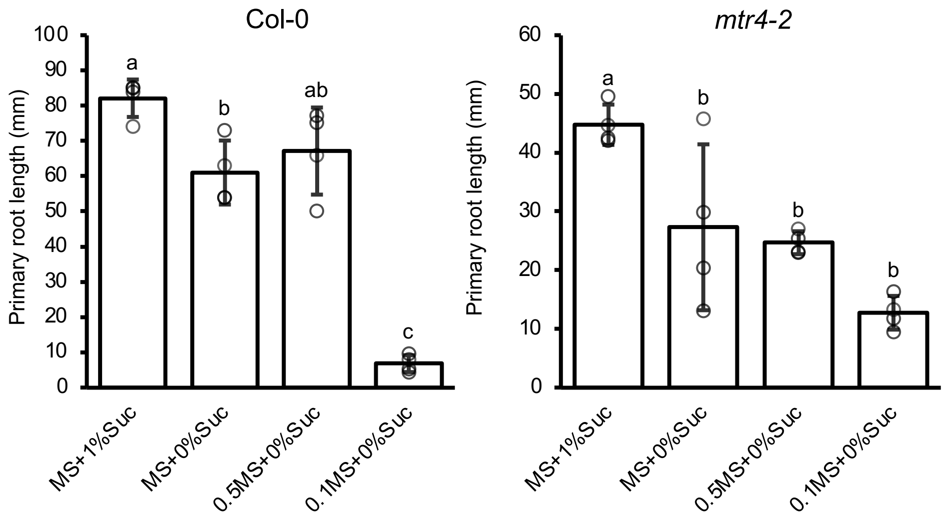

Supplementary Figure S2. Primary root lengths of 14-day-old seedlings of the wild type (Col-0) and *mtr4-1* grown under different nutrient conditions. MS+1%Suc, full-strength MS medium with 1% (w/v) Suc, MS+0%Suc; full-strength MS medium without Suc; 0.5MS+0%Suc, half-strength (0.5×) MS medium without Suc; 0.1MS+0%Suc, one tenth–strength (0.1×) MS medium without Suc. Values are expressed as means ± standard deviation (SD; n=4). Different lowercase letters indicate significant differences (Tukey-Kramer test, p<0.05).

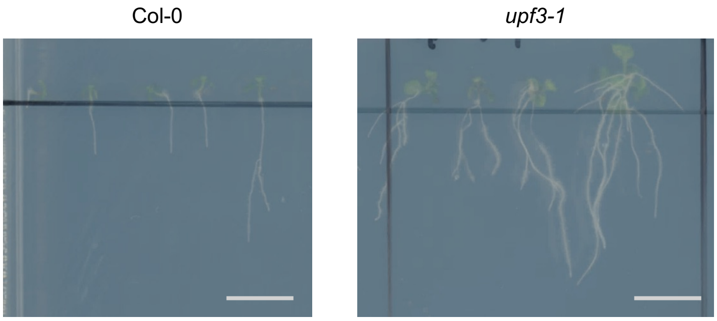

Supplementary Figure S3. Representative photographs of 14-day-old seedlings of the wild type (Col-0) and the *upf3-1* mutant grown on medium containing 0.1 × MS without sucrose. Scale bars, 1 cm.

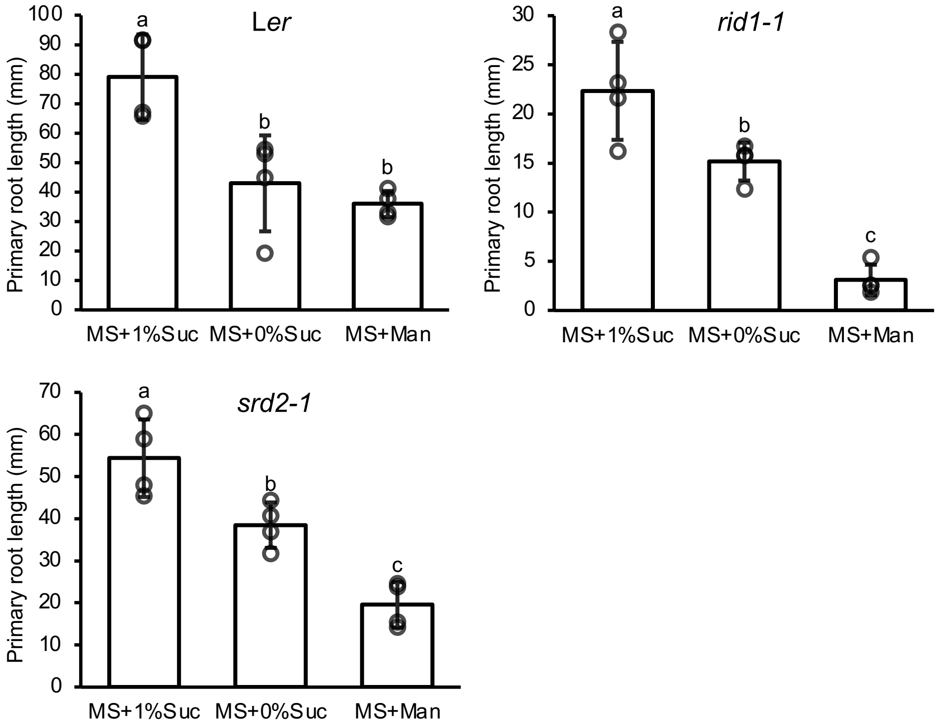

Supplementary Figure S4. Primary root lengths of 14-day-old seedlings of the wild type (L*er*) and the *rid1-1* and *srd2-1* mutants grown under different nutrient conditions. MS+1%Suc, full-strength MS medium with 1% (w/v) Suc; MS+0%Suc; full-strength MS medium without Suc; MS+Man; full-strength MS medium with 0.75% (w/v) Man. Values are expressed as means ± standard deviation (SD; n=4). Different lowercase letters indicate significant differences (Tukey-Kramer test, p<0.05). The experiments were repeated more than three times and the tendency of the results was confirmed. The results for one replicate are shown.

A

Scale	1	0.8	0.6	0.4	0.2	0	-0.2	-0.4	-0.6	-0.8	-1

		-N	-K	-Ca	-Mg	-P	-S	-B	-Fe	-Mn	-Zn	-Cu	-Mo
CCR4A	AT3G58560	0.06072	0.12143	0.01491	0.10992	0.0226	0.07175	0.07275	0.09824	0.10829	0.06151	0.11229	0.06169
CCR4B	AT3G58580	0.23907	0.04112	0.13108	0.09489	0.14136	0.08326	0.04104	0.09618	0.10152	0.07178	0.09226	0.0266
MTR4	AT1G59760	-0.2823	-0.0734	0.07549	-0.0825	-0.1447	-0.003	0.09612	0.03342	-0.0054	-0.0767	0.03562	-0.0193
UPF3	AT1G33980	0.14962	0.09032	-0.0429	0.04875	-0.0094	0.01287	0.00691	0.00484	0.07145	0.06904	-0.0233	0.0213
RFC3	AT3G17170	-1.0092	-0.1351	0.20415	-0.1541	-0.3534	-0.1555	-0.0212	-0.1005	0.05551	-0.1016	-0.0577	-0.0159
RID1	AT1G26370	0.02429	0.18357	0.08473	0.04932	-0.099	-0.0293	0.15721	0.06178	0.06061	0.03806	-0.0181	0.13785
SRD2	AT1G28560	-0.0089	0.0543	-0.0285	-0.0771	0.00126	0.09542	0.31031	0.04005	0.02296	-0.154	-0.0094	0.03289

B

		Relative abundance (%)					
		First dataset		Second dataset		q value	
Gene	Isoform	Control	Treated	Control	Treated	First dataset	Second dataset
UPF3	AT1G33980.1	99.9982742	99.2540103	99.9983536	99.2987122	0.003835389	0.0001612
	AT1G33980.2	0.00172582	0.74598966	0.00164643	0.70128782		

Supplementary Figure S5. Changes in expression levels and alternative splicing of *CCR4A*, *CCR4B*, *MTR4*, *UPF3*, *RFC3*, *RID1*, and *SRD2* in response to the depletion of different nutrients. (A) Relative expression levels of the genes assessed in this study in response to the depletion of the indicated nutrient. The expression data (log_2[fold change] between the mock and nutrient depletion conditions) were obtained from Nishida et al. (2017) and visualized as a heatmap. No changes were statistically significant. (B) Changes in the abundance of mRNA isoforms of *UPF3* in response to nitrogen depletion (Nishida et al. 2017). The relative abundances of each alternative splicing isoform of *UPF3* (*AT1G33980.1* and *AT1G33980.2*) are shown.

Nishida S, Kakei Y, Shimada Y, Fujiwara T (2017) Genome-wide analysis of specific alterations in transcript structure and accumulation caused by nutrient deficiencies in *Arabidopsis thaliana*. *Plant J* 91: 741-753
